# Pharmacogenomics: The Right Drug to the Right Person

**DOI:** 10.4021/jocmr2009.08.1255

**Published:** 2009-10-16

**Authors:** Aneesh T P, Sonal Sekhar M, Asha Jose, Lekshmi Chandran, Subin Mary Zachariah

**Affiliations:** a Amrita School of Pharmacy, Amrita Vishwavidyapeetham University, Kochi-682026, Kerala, India

## Abstract

Pharmacogenomics is the branch of pharmacology which deals with the influence of genetic variation on drug response in patients by correlating gene expression or single-nucleotide polymorphisms with a drug's efficacy or toxicity. It aims to develop rational means to optimize drug therapy, with respect to the patients genotype, to ensure maximum efficacy with minimal adverse effects. Such approaches promise the advent of personalized medicine, in which drugs and drug combinations are optimized for each individual's unique genetic makeup. Pharmacogenomics is the whole genome application of pharmacogenetics, which examines the single gene interactions with drugs.

**Keywords:**

Pharmacogenetics; Single nucleotide polymorphisms; Genomics; Genotype

## Introduction

Pharmacogenomics is the study of how an individual's genetic inheritance affects the body's response to drugs. The term comes from the words pharmacology and genomics and is thus the intersection of pharmaceuticals and genetics. Pharmacogenomics holds the promise that drugs might one day be tailor-made for individuals and adapted to each person's own genetic makeup. Environment, diet, age, lifestyle, and state of health all can influence a person's response to medicines, but understanding an individual's genetic makeup is thought to be the key to creating personalized drugs with greater efficacy and safety. The way a person responds to a drug (this includes both positive and negative reactions) is a complex trait that is influenced by many different genes. Without knowing all of the genes involved in drug response, scientists have found it difficult to develop genetic tests that could predict a person's response to a particular drug [1]. Once scientists discovered that people's genes show small variations (or changes) in their nucleotide (DNA base) content, all of that changed: genetic testing for predicting drug response is now possible. Pharmacogenomics combines traditional pharmaceutical sciences such as biochemistry with annotated knowledge of genes, proteins, and single nucleotide polymorphisms.The most common variations in the human genome are called single nucleotide polymorphisms (SNPs). There is estimated to be approximately 11 million SNPs in the human population, with an average of one every 1,300 base pairs.

## History

Genomics was established by Fred Sanger when he first sequenced the complete genomes of a virus and a mitochondrion. His group established techniques of sequencing, genome mapping, data storage, and bioinformatic analyses in the 1970-1980s. The actual term genomics is thought to have been coined by Dr. Tom Roderick, a geneticist at the Jackson Laboratory (Bar Harbor, ME) over beer at a meeting held in Maryland on the mapping of the human genome in 1986.

In 1972, Walter Fiers and his team at the Laboratory of Molecular Biology of the University of Ghent (Ghent, Belgium) were the first to determine the sequence of a gene: the gene for Bacteriophage MS2 coat protein. In 1976, the team determined the complete nucleotide-sequence of bacteriophage MS2-RNA. The first DNA-based genome to be sequenced in its entirety was that of bacteriophage Φ-X174 (5,368 bp), sequenced by Frederick Sanger in 1977 [2,3].

The first free-living organism to be sequenced was that of Haemophilus influenzae in 1995, and since then genomes are being sequenced at a rapid pace. A rough draft of the human genome was completed by the Human Genome Project in early 2001, creating much fanfare.

As of September 2007, the complete sequence was known of about 1879 viruses, 577 bacterial species and roughly 23 eukaryote organisms, of which about half are fungi. Most of the bacteria whose genomes have been completely sequenced are problematic disease-causing agents, such as Haemophilus influenzae. Pharmacogenomics combines traditional pharmaceutical sciences such as biochemistry with annotated knowledge of genes, proteins, and single nucleotide polymorphisms[4,5].

## Importance of pharmacogenomics

Adverse Drug Reaction conveys little of the horror of a severe negative reaction to a prescribed drug. But such negative reactions can nonetheless occur. A 1998 study of hospitalized patients published in the Journal of the American Medical Association reported that in 1994, adverse drug reactions accounted for more than 2.2 million serious cases and over 100,000 deaths, making adverse drug reactions (ADRs) one of the leading causes of hospitalization and death in the United States [6]. For instance, the daily doses required to treat patients vary by 20-fold for the warfarin, by 40-fold for the antihypertensive drug propranolol and by 60-fold for L-dopa for parkinsons disease. Other drugs have clinical utility in a subset of patients with given pathology, e.g., antipsychotics that are ineffective in 30% of schizophrenics, suggesting that such drugs are only effective in patients with specific disease etiologies [7]. Many of the deaths could be avoided if the physician had prior knowledge of patients genetic profile, which determines the drug response. Currently, there is no simple way to determine whether people will respond well, badly, or not at all to a medication; therefore, pharmaceutical companies are limited to developing drugs using a one size fits all system [8]. This system allows for the development of drugs to which the average patient will respond. But, as the statistics above show, one size does not fit all, sometimes with devastating results. What is needed is a way to solve the problem of ADRs before they happen. The solution is in sight though, and it is called pharmacogenomics.

Pharmacogenomics eventually can lead to an overall decrease in the cost of health care because of decreases in: (1) the number of adverse drug reactions; (2) the number of failed drug trials; (3) the time it takes to get a drug approved; (4) the length of time patients are on medication; (5) the number of medications patients must take to find an effective therapy; (6) the effects of a disease on the body (through early detection) [9].

## Pharmacogenomics today

The cytochrome P450 (CYP) family of liver enzymes is responsible for breaking down more than 30 different classes of drugs. DNA variations in genes that code for these enzymes can influence their ability to metabolize certain drugs. Less active or inactive forms of CYP enzymes that are unable to break down and efficiently eliminate drugs from the body can cause drug overdose in patients. Today, clinical trials researchers use genetic tests for variations in cytochrome P450 genes to screen and monitor patients. In addition, many pharmaceutical companies screen their chemical compounds to see how well they are broken down by variant forms of CYP enzymes [10].

Another enzyme called TPMT (thiopurine methyltransferase) plays an important role in the chemotherapy treatment of a common childhood leukemia by breaking down thiopurines. A small percentage of Caucasians have genetic variants that prevent them from producing an active form of this protein. As a result, thiopurines elevate to toxic levels in the patient because the inactive form of TMPT is unable to break down the drug. Today, doctors can use a genetic test to screen patients for this deficiency, and the TMPT activity is monitored to determine appropriate thiopurine dosage levels [11].

## Pharmacogenomics in future

New developments in this field will impact on drug design at three main levels: (1) the interaction of the drug with its receptor binding site; (2) the absorption and distribution of the drug; (3) the elimination of the drug from the body.

## Benefits of pharmacogenomics

Pharmacogenomics combines traditional pharmaceutical sciences such as biochemistry with annotated knowledge of genes, proteins, and single nucleotide polymorphisms. Following are the benefits.

### More powerful medicines

Pharmaceutical companies will be able to create drugs based on the proteins, enzymes, and RNA molecules associated with genes and diseases. This will facilitate drug discovery and allow drug makers to produce a therapy more targeted to specific diseases. This accuracy will not only maximize therapeutic effects but also decrease damage to nearby healthy cells.

### Better, safer drugs the first time

Instead of the standard trial-and-error method of matching patients with the right drugs, doctors will be able to analyze a patient's genetic profile and prescribe the best available drug therapy from the beginning. Not only will this take the guesswork out of finding the right drug, it will speed recovery time and increase safety as the likelihood of adverse reactions is eliminated.

### More accurate methods of determining appropriate drug dosages

Current methods of basing dosages on weight and age will be replaced with dosages based on a person's genetics; how well the body processes the medicine and the time it takes to metabolize it. This will maximize the therapy's value and decrease the likelihood of overdose.

### Advanced screening for disease

Knowing one's genetic code will allow a person to make adequate lifestyle and environmental changes at an early age so as to avoid or lessen the severity of a genetic disease. Likewise, advance knowledge of particular disease susceptibility will allow careful monitoring, and treatments can be introduced at the most appropriate stage to maximize their therapy.

### Better vaccines

Vaccines made of genetic material, either DNA or RNA, promise all the benefits of existing vaccines without all the risks. They will activate the immune system but will be unable to cause infections. They will be inexpensive, stable, easy to store, and capable of being engineered to carry several strains of a pathogen at once.

### Improvements in the drug discovery and approval process

Pharmaceutical companies will be able to discover potential therapies more easily using genome targets. The drug approval process should be facilitated as trials are targeted for specific genetic population groups and providing greater degrees of success. The cost and risk of clinical trials will be reduced by targeting only those persons capable of responding to a drug.

### Decrease in the overall cost of health care

Decreases in the number of adverse drug reactions, the number of failed drug trials, the time it takes to get a drug approved, the length of time patients are on medication, the number of medications patients must take to find an effective therapy, the effects of a disease on the body (through early detection), and an increase in the range of possible drug targets will promote a net decrease in the cost of health care [12].

## Barriers to pharmacogenomics progress

Pharmacogenomics is a developing research field that is still in its infancy. Several of the following barriers will have to be overcome before many pharmacogenomics benefits can be realized. They are the followings.

### Complexity of finding gene variations that affect drug response

Single nucleotide polymorphisms (SNPs) are DNA sequence variations that occur when a single nucleotide (A, T, C, or G) in the genome sequence is altered. SNPs occur every 100 to 300 bases along the 3-billion-base human genome, therefore millions of SNPs must be identified and analyzed to determine their involvement (if any) in drug response. Further complicating of the process is our limited knowledge of which genes are involved with each drug response. Since many genes are likely to influence responses, obtaining the big picture on the impact of gene variations is highly time-consuming and complicated.

### Limited drug alternatives

Only one or two approved drugs may be available for treatment of a particular condition. If patients have gene variations that prevent them using these drugs, they may be left without any alternatives for treatment.

### Disincentives for drug companies to make multiple pharmacogenomic products

Most pharmaceutical companies have been successful with their one size fits all approach to drug development. Since it costs hundreds of millions of dollars to bring a drug to market, will these companies be willing to develop alternative drugs that serve only a small portion of the population [13]?

## Impact on pharmacy profession

Presently doctors diagnose and prescribe a drug on the trial and error basis and pharmacist advices about side effects and drug-drug interaction. But a day will come when you will take a gene report instead of blood reports .Thus after the diagnosis, pharmacist would interpret the panels of genetic results and advice you which drug would be best for your particular gene so that you have fast recovery.

## Conclusions

Pharmacogenomics in pharmaceutical industry is a potential tool, awaiting use for the maximum benefit. It represents a radical advance in medical history. The main aims of it are; personalized therapy, improvement in efficacy and reduction in adverse drug reactions, correlation of genotype with clinical genotype, identification of novel targets for new drugs, and pharmacogenetic profiling of patients to predict disease susceptibility and drug response. In the past, most drugs were designed to work on the population level rather than being targeted for the individual patient. By reversing that trend, pharmacogenomics helps to refine the focus of treatment and makes drugs more effective and less toxic. Rather than relying on the outward manifestation of disease the signs and symptoms that physicians call the phenotype pharmacogenomic medicine examines and treats the genotype. Gradual inclusion of pharmacogenomic studies in drug discovery and development will cause substantial reduction in the expenses involved in drug development, ensure a safe clinical trial and reduce failures. Thus, many potential drugs which may be lost due to the effects on the outliers in a study can be retained when pharmacogenomic study is used in the future.
